# Props and Buttresses

**Published:** 1890-10-04

**Authors:** 


### THE DOCTOR'S PULPIT.

### PROPS AND BUTTRESSES.

THE solidest building will not stand for ever; but the most ricketty affair will stand a long time when it is judiciously propped and buttressed. Mistress Nature seems anything but impartial in her distribution of constitutions to men and women. Here, for example, is a country squire who can get the best of everything to eat, and live out of doors all day without fatigue or danger from exposure to the changeable weather. He might very well have afforded to be born a little weakly, because every circumstance of his life, from the cradle to the grave, may be made to conduce to the purifying of his blood and the building up of his strength. But Nature, as if resolved to "carry coals to Newcastle," has endowed him with a constitution that an elephant might envy. There, on the other hand, is a poor man, who is doomed to earn his living on a tailor's shop-board. To stand his long hours and the stuffy atmosphere which he daily has to breathe he needs lungs of leather and the robustness of a prize-fighter. Instead of these, Nature gave him at the outset a feeble frame and a sickly constitution; and placed him where he could only be half fed, ill-clothed, and miserably lodged; so that every circumstance of his existence has seemed to conspire with every other to puff out the originally feeble flame of his life, without giving him a single chance of getting strength and brightness. There may be "law" in these things, as, indeed, there undoubtedly is, but the "justice" of them is not easy to discover. That there is justice as well as law we do not doubt; but we are not inclined to be very severe on the constitutionally feeble man who repines sometimes, and wonders whether Providence either knows or cares about his troubles.

There are people so feebly born that they require to be propped from their earliest days; there are others who, though born strong, have been so buffeted about by the storms of life that they, too, if they are to stand much longer, need to be well buttressed, and put into thorough repair. Now, there is a difference between a man and a church, or a house, or a stable. If the house or the stable get out of repair, it has an owner who is bound to see to it, and who will suffer in his pocket if he neglect to do so. But men have no owners to order repairs to be made when they are falling into decay. Women and children have, perhaps; but not men. A man is generally the owner of himself, and it is for him to see when and in what way he is out of repair, and to give orders to the physiological builder to build him up and put him into good order, and to apply to him such props and buttresses as may make him stand against wind and weather as long as possible. Very few people seem to have the sense to understand that "a pill in time saves nine." Is anybody so destitute of imagination as not to see that the word "pill" here does not stand for a little round pellet of aperient drugs encased in sugar or silver paper, but for skilled medical treatment in general? It ought to be quite easy to understand that just as a tile put on at the proper time may save the whole roof, or a judicious brick may keep the wall from falling, so may a little proper medical treatment at an early stage restore health and make long life sure. The doctor often sees this, and sees it very clearly. But then he has no proprietary right in the patient; he cannot go and say, "Sir, or madam, you are getting out of repair, and need a brick in your physiological wall, or a tile on the roof of your health." Perhaps it would be a very good thing if the doctor could sometimes use such freedom with friends or even with strangers. But there is nothing more certain than that he cannot. Every priestly confessor of medical etiquette would inflict upon him the greater excommunication at once if he did; and even the person to be benefited by his advice would hold him to be guilty of the worst possible taste.

So, then, if every man is to keep his physiological house weather-proof he must be his own architect and surveyor. He alone is responsible for the repairs, and if he be at all a wise man he will constantly act upon the lines of the "tile in time" theory. Everybody admits that the practical builder ought to understand his business; and few will deny that it is desirable for the architect and surveyor also to have a little knowledge of the matter in hand. The architect and surveyor, in the question under discussion, is, as we have said, the out-of-repair man himself. There is no doubt, whatever anybody may think or say to the contrary, that every man ought to have as much knowledge of physiology and medicine as to enable him to understand when it would be desirable to call in a doctor. It is as certain as anything can be that intelligent doctoring keeps thousands of people alive to a good old age who would otherwise die early. It cannot be denied that nature has bestowed upon many of us feeble constitutions. We are physiological buildings placed on a bad foundation, and built of soft stone or bad bricks and poor mortar. In the case of many people, Nature has been a "jerry builder." There is no use denying it. All the fine-spun theories in the world about the perfection of Nature's work will not alter the hard facts. But if that be so, can art mend Nature's work? Can an honest builder improve a jerry builder's house? Undoubtedly. By clever props and well-applied buttresses he can make almost the worst wall that ever was built stand for a long time. Exactly so can the judicious doctor prop and buttress up a feeble constitution and miserable health. Give but the doctor the same chance as the builder has; that is to say, put the bad constitution and the feeble health entirely under his charge, and he will be a very bungling practitioner if he cannot do something to make the house stand a little longer.

Are we entering a plea for more work for the doctor? Nonsense, Mr. Cynic! If you have not the brains to understand all the experienced philosophy which lies in a "stitch in time," and in "every man to his trade," you are but a stupid fellow, however sarcastically you may talk! Our whole sermon is built up on these two texts. You will not deny that a stitch in time is a good rule. Nor will you doubt that every man to his trade is equally safe in practice. It is very evident that many people, either from having an originally weak constitution, or as the result of the wear and tear of life, are in a battered and shaky condition. It is equally obvious that they will be wise to get themselves propped and buttressed up. Who is so fit and proper to do the work as the man whose trade is that of a physiological builder?

